# Visualizing the Cellular and Subcellular Distribution of Fms-like Tyrosine Kinase 3 (Flt3) and Other Neuronal Proteins Using Alkaline Phosphatase (AP) Immunolabeling

**DOI:** 10.3390/ijms26052284

**Published:** 2025-03-04

**Authors:** Yuqin Yin, Kathleen Z. He, Jane Kirby, Ishraq A. Haque, Xin Tang

**Affiliations:** 1 Department of Neurosurgery, Boston Children’s Hospital, 300 Longwood Ave, Boston, MA 02115, USA; 2 F. M. Kirby Neurobiology Center, Boston Children’s Hospital, 3 Blackfan Circle, Boston, MA 02115, USA; 3 Department of Neurosurgery, Harvard Medical School, Boston, MA 02115, USA; 4 Harvard College, Cambridge, MA 02138, USA

**Keywords:** Flt3, brain development, AP-polymer histochemistry, weakly expressed proteins, histochemistry and immunofluorescent co-staining, Kir2.1, PSD95, human stem-cell derived neurons

## Abstract

Precisely localizing the spatial distribution of proteins within various brain cell types and subcellular compartments, such as the synapses, is essential for generating and testing hypotheses to elucidate their roles in brain function. While the fms-like tyrosine kinase-3 (Flt3) has been extensively studied in the context of blood cell development and leukemia pathogenesis, its role in the brain remains poorly understood. Previous efforts to address this issue were hindered by the low expression levels of Flt3 and the limited sensitivity of the standard immunolabeling method, which were insufficient to reliably detect Flt3 protein in brain tissue. In this study, we systematically characterized Flt3 protein localization during brain development using a highly sensitive immunolabeling method based on alkaline phosphatase (AP) polymer biochemistry. This approach revealed a previously unrecognized neuron-selective Flt3 expression pattern in both mouse and human cerebella, with a developmental increase in total protein levels accompanied by a shift from a cytosolic to a dendritic subcellular distribution. Combining AP-polymer-based immunohistochemistry (AP-IHC) for Flt3 with conventional immunostaining of cell type marker proteins revealed parvalbumin- and calbindin-positive Purkinje cells to be the main cell type expressing Flt3 in the cerebellum. To validate the versatility of the AP-IHC method for detecting low-abundance neuronal proteins, we demonstrated robust labeling of Kir2.1, a potassium channel protein, in brain tissue sections from mouse, pig, and human samples. We further applied the AP-IHC method to human stem cell-derived neurons, effectively visualizing the postsynaptic density scaffold protein PSD95 within synapses. To our knowledge, this is the first study to employ an AP-IHC method combined with other standard immunofluorescent staining to co-detect weakly expressed neuronal proteins and other cellular markers in brain tissue and cultured neurons. Additionally, our findings uncover a previously unrecognized neuron-specific pattern of Flt3 expression in the cerebellum, laying the foundation for future mechanistic studies on its role in normal brain development and neurological disorders.

## 1. Introduction

The brain expresses the majority of known protein-coding genes [1]. In many cases, the cellular expression and subcellular distribution of their protein products—including signaling receptors, ion channels, transporters, and synaptic proteins—remain poorly characterized. One such example is the receptor tyrosine kinase Flt3, which is critical for blood cell development and is implicated in the pathogenesis of leukemia when dysregulated [2]. While the presence of Flt3 mRNA in the brain has been reported, the Flt3 protein expression in the brain lacks rigorous investigation [3,4]. Single-cell sequencing studies of mouse and human brain tissue reveal high Flt3 mRNA expression in the cerebellum, particularly in Purkinje cells [5], yet the extent to which Flt3 protein expression correlates with its mRNA level within these cells remains unknown. Moreover, the subcellular localization of the Flt3 protein is a crucial determinant of signaling strength and mode [6], but cannot be inferred from RNA sequencing data. Our group’s previous unbiased drug screening efforts discovered that pharmacological inhibition of Flt3 kinase signaling in brain cells enhances the expression of KCC2—a neuron-specific chloride transporter protein which is essential for GABAergic inhibition and normal brain development [7]. These findings suggest an unexpected role of Flt3 signaling in neurons and underscore the need for sensitive in situ protein detection methods to study its disposition in brain tissue comprehensively.

Towards this end, we sought to develop an immunolabeling method with high sensitivity and resolution to investigate Flt3 expression patterns in neurons. Standard fluorescent immunostaining method primarily relies on fluorescent secondary antibodies which are large proteins with limited binding affinity, signal amplification, and sensitivity [8]. One alternative method often used is Horseradish peroxidase (HRP)-based histochemistry, where HRP conjugated to a secondary antibody via avidin/biotin system catalyzes an in situ reaction to oxidize the chromogenic substrate, resulting in robust signal amplification [9]. However, this method produces a non-fluorescent, monochromatic deposit that interferes with fluorescent light emission, making it unsuitable for simultaneous staining with other target proteins, such as cell type or subcellular organelle markers, labeled by fluorescent antibodies [10]. To overcome this limitation, we found that the alkaline phosphatase polymer histochemistry technique (AP-IHC), invented by Vector Laboratories, was a highly sensitive staining method that could be used to develop a co-staining method with other fluorescent antibodies. AP-IHC employs secondary antibodies conjugated to multiple copies of alkaline phosphatase, enabling in situ deposition of fluorescent products for high-sensitivity protein labeling. Although AP-IHC has been used in histological analysis [11], it has not been applied to immunolabeling in the brain tissue, which presents unique challenges. These challenges include the brain’s complex cellular composition, high lipid content, and the presence of fine structures such as synapses where certain proteins are concentrated. Therefore, it is important to explore a strategy to apply the AP-IHC method in brain studies.

In this study, we developed an optimized workflow for applying the highly sensitive AP-IHC in brain tissue and, more importantly, introduced a hybrid method that combines AP-IHC with the conventional immunofluorescent staining, enabling multiplexed in situ protein labeling. Using this hybrid approach, we systematically investigated Flt3 expression during cerebellar development alongside various proteins essential for neuronal functions. Our work provides the first demonstration of the versatility and sensitivity of AP-IHC for detecting a broad range of antigens in brain cells with cellular and subcellular resolution. Through direct comparisons with standard Alexa Fluor-conjugated antibodies and HRP-based histochemistry, we show that AP-IHC offers superior sensitivity and specificity for visualizing Flt3 kinase in both developing and adult mouse brain samples. This sensitivity, combined with the compatibility of AP-IHC co-staining with traditional immunofluorescence, enables the discovery of a neuron-specific pattern of Flt3 expression in brain tissues. We also show that AP-IHC successfully detects Kir2.1, an inward-rectifying potassium channel expressed at low levels, in mouse, pig, and human brain tissue samples. In addition, we extended using AP-IHC to label neuronal antigen PSD95, enriched in microscopic subcellular synaptic structures, in human stem cell-derived neurons. These results establish AP-IHC as a powerful and versatile tool for high-sensitivity, high-resolution in situ detection of brain proteins, facilitating detailed investigation of cellular and synaptic protein distributions in tissue or cell culture preparations across multiple species.

## 2. Results

### 2.1. Establish the Spatial–Temporal Pattern of Flt3 Expression During Brain Development Using AP Immunolabeling

**Figure 1:**
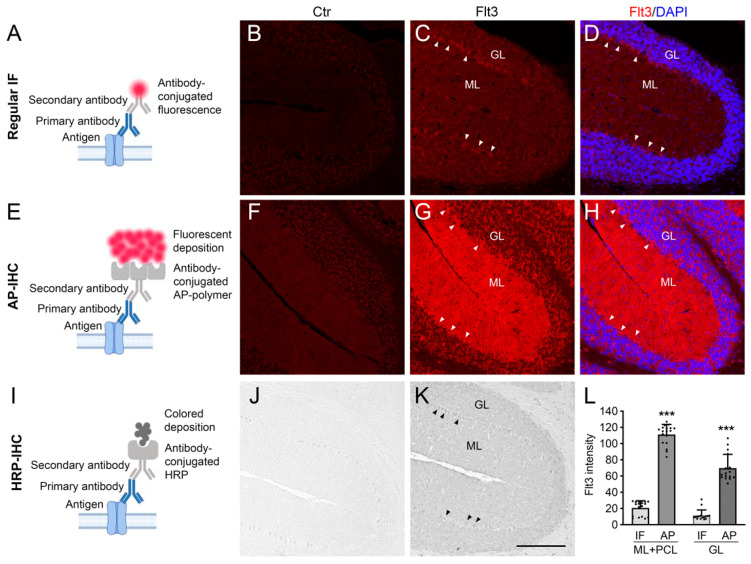
Direct comparison of different staining methods for visualizing the Flt3 protein in brain tissue. Adult mouse cerebellum sagittal sections (cryostat, 10 µm in thickness), were immunostained by three different methods in order to visualize a weakly expressed protein Flt3. After antigen retrieval, in a regular immunofluorescent staining (Regular IF in (**A**–**D**); IF in (**L**)), Flt3 was rarely seen in both the molecular layer (ML) and granular layer (GL), with weak signals in the Purkinje cell layer (arrow heads in images, PCL in (**L**)). In contrast, with AP-polymer-based histochemistry (AP-IHC in (**E**–**H**), AP in (**L**)), Flt3 was visualized by strong fluorescent signals, with the intensity > 5-fold higher than that from the regular IF in the ML+PCL area, almost 7-fold higher in GL area (**L**). The HRP-based histochemistry method (HRP-IHC in (**I**–**K**)) showed clear positive signals, but the staining was unable to show the intensity differences between ML and GL. In all 3 staining methods, the staining controls (Ctr) were processed exactly the same, except applying the same amount of rabbit IgG, replacing the Flt3 rabbit primary antibody (Flt3). Scale bar: 200 µm. Mean ± SD. *** *p* < 0.001 (AP vs. IF). One-way ANOVA followed by Bonferroni test.

To lay the groundwork for mechanistic studies of Flt3 signaling in the brain, we set out to define the expression pattern of Flt3 in brain tissue. Our focus was on the cerebellum, as previous studies have reported enriched Flt3 mRNA expression in this region [5]. For this, we adapted the AP-IHC method, traditionally used in peripheral tissue analysis [11], for use in brain tissue. Compared to regular immunofluorescent staining (IF) with a secondary fluorescent antibody that failed to produce a consistent Flt3 expression pattern (Figure 1A–D), the AP-IHC method successfully highlighted the Flt3 protein with bright fluorescence in mouse cerebellum slices (Figure 1E–H). The staining pattern closely resembled the results obtained using the HRP method, which produces a monochromatic deposit (Figure 1I–K). Notably, the AP-IHC method revealed distinct Flt3 expression differences between the cerebellar granular layer (GL) and molecular layer (ML)—distinctions that were less discernible with the HRP method. Quantitative analysis confirmed that the AP-IHC method significantly improved Flt3 labeling sensitivity and signal-to-noise ratio, achieving a five- to seven-fold higher staining intensity compared to conventional immunofluorescence (Figure 1L). These findings demonstrate the enhanced capability of AP-IHC for investigating the spatial–temporal patterns of Flt3 expression during brain development.

**Figure 2:**
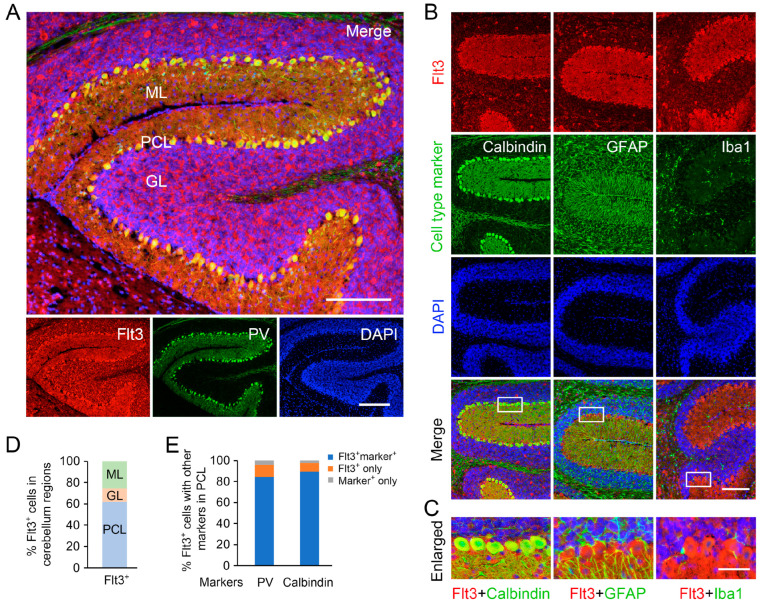
Flt3 is expressed specifically in neurons. We created a hybrid method by combining AP-IHC with regular IF to visualize the cell types and intracellular localization of the Flt3 protein. Postnatal D14 mouse brain sections were used for the staining. Flt3 was visualized by AP-IHC (red signal in (**A**–**C**)) and other cell markers were visualized by regular IF staining (green). (**A**–**C**), Flt3 was co-localized with the inhibitory neuronal markers parvalbumin (PV) and calbindin in the Purkinje cell layer (PCL) which are NeuN-negative (see Figure 4A), but not with the glial cell marker GFAP or the microglial cell marker Iba1. Besides the cell body, Flt3 is enriched in dendrites of Purkinje cells as well as in the subsets of small cells in the molecular layer (ML) and some large cells in the granular layer (GC) that are NeuN-positive (Supplementary Figure S1). The % of Flt3^+^ cells is about 60% in PCL, 13% in GL, and 25% in ML, respectively (**D**). In PCL, about 85% of cells are Flt3- and PV-double-positive, 4% are PV-positive but Flt3-negative (PV^+^ only), and 11% are Flt3-positive and PV-negative (Flt3^+^ only, **E**); about 90% cells are Flt3_-_ and calbindin-double-positive (**E**). None of the Flt3^+^ cells are GFAP^+^ or Iba1^+^. Scale bars in (**A**): upper panel: 280 µm; lower panel: 220 µm; (**B**): 200 µm, (**C**): 50 µm.

Among the advantages of the AP-IHC method, as we described above, a unique feature is its ability to produce fluorescent signals in the Cy3 channel without interfering with other fluorescent channels. This capability enables multiplexed co-labeling of various antigens when combined with the conventional fluorescent immunostaining. Using a hybrid protocol developed through fine-tuning detergent usage, we uncovered a previously uncharacterized pattern of Flt3 protein expression in mouse cerebellar tissue. Our results reveal that Flt3 expression is restricted to neurons, with no overlap observed in GFAP^+^ astrocytes or Iba1^+^ microglia (Figure 2). Interestingly, Flt3 expression is particularly enriched in Purkinje cells (PCL), which co-express parvalbumin (PV) or calbindin (Figure 2), whereas NeuN^+^ granular cells show only a limited level of Flt3 staining (Supplementary Figure S1).

**Figure 3:**
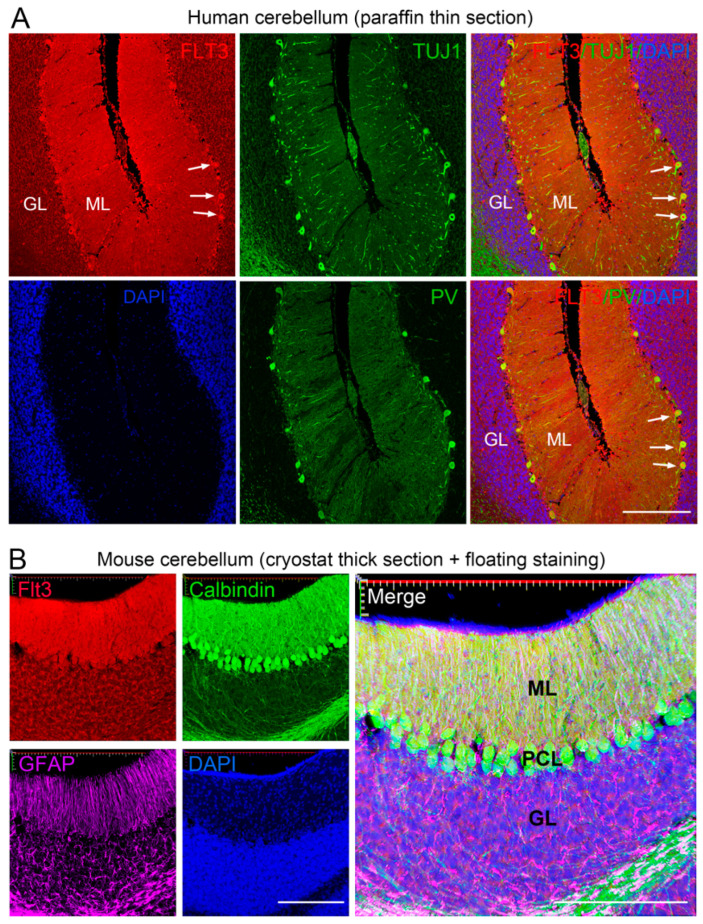
AP-IHC hybrid staining method shows comparable performance in various brain tissue sample conditions. (**A**). Human cerebellar tissue, after years of prolonged formalin fixation, was paraffin-embedded, sectioned, and stained by the FLT3 AP-IHC (red), followed by regular immuno-fluorescent staining (green) for either βIII tubulin (antibody TUJ1), a pan-neuronal marker, or parvalbumin (PV), a marker of inhibitory neurons. Similar to the pattern in mouse brains, FLT3 is enriched in Purkinje cells (arrows) and in the molecular layer (ML), with much lower levels in the granular layer (GL). (**B**). Stacked confocal images show free-floating staining of mouse brain slices with the hybrid labeling method and the images were taken from the Zeiss LSM 980 airyscan 2 confocal microscope (10× object, Zeiss Microscopy LLC, Oberkochen, Germany). Postnatal D14 mouse cerebellum slices (40 µm in thickness) were used for Flt3 AP-IHC (red) with calbindin (green) and GFAP (purple) triple staining. Flt3 is well-localized in calbindin^+^ neurons and their processes but not in GFAP astrocytes. Scale bars in (**A**): 300 µm; in (**B**): both, 200 µm.

We further validated Flt3 expression in neurons, particularly in the cerebellum inhibitory neurons, in human brain tissue. Using the AP-IHC hybrid staining method, we successfully detected Flt3 in postnatal human cerebellum sections that had been preserved in formalin for several years. Similar to that in mouse cerebellum tissues, FLT3 is enriched in human Purkinje cells and their dendrites in the molecular layer (Figure 3A). FLT3 staining is co-localized with the general neuronal marker TUJ1 (for detecting β-III tubulin) and the inhibitory neuronal marker parvalbumin (PV) (Figure 3A). In the Purkinje cell layer, about 98% FLT3^+^ cells are TUJ1^+^, and 95% are PV^+^. Beside the thin sections from cryostat (10 µm in Figure 1 and Figure 2), or paraffin-embedded (5 µm in Figure 3A) samples, the AP-IHC hybrid method can be applied for floating staining of thick mouse brain slices (40 µm, Figure 3B), which are commonly used in brain studies [12]. To confirm that the Flt3 staining is not only restricted to the tissue surface but is well distributed throughout the entire thickness of the section, we used confocal microscopy to examine the staining. Our analysis revealed that Flt3 AP-IHC immunoreactivity is evenly distributed across a series of confocal scanned sections (Supplementary Figure S2). Consistent with staining in thin sections, the deposition from Flt3 AP-IHC in thick free-floating mouse brain sections does not interfere with other co-stained markers (Figure 3B, Supplementary Figure S2).

**Figure 4:**
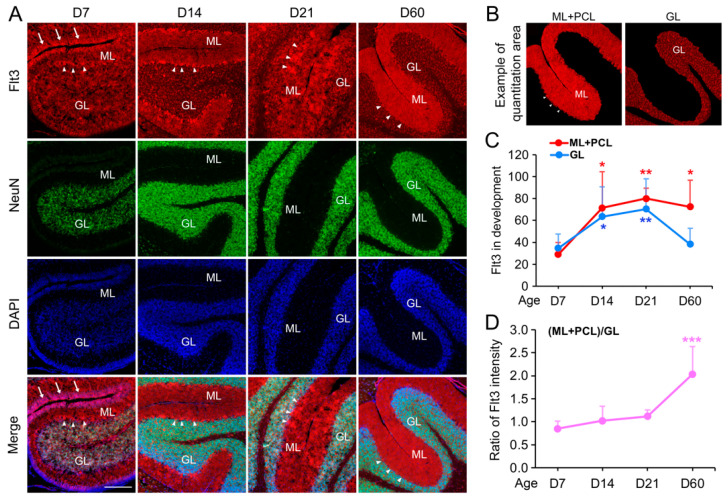
The developmental upregulation and shifted localization of Flt3 in the cerebellum. C57 mouse brains were harvested at postnatal D7, D14, D21, and D60, and cryostat sections (10 µm in thickness) were co-stained for Flt3 and NeuN with the hybrid staining method (**A**). At D7, Flt3 was expressed at the low level both in ML, GL, PCL (arrow heads), and in the epineurium layer that was NeuN-negative (arrows). At D14, Flt3 expression dramatically increased in both GL and in (ML+PCL) (**A**,**C**), and most Purkinje cells showed Flt3 immunoreactivity in both cell body and dendrites ((**A**) and in Figure 2A–C and Figure 3B). At D21, strong Flt3 immunoreactivity started to shift from GL to ML, and at D60, most of the Flt3 staining was enriched in ML (**A**). The shift was substantial and dramatic, showing as the ratio of (ML+PCL)/GL (**D**). (**B**) is an example of the areas defined for quantitation. Scale bar in (**A**): 220 µm. Mean ± SD, * *p* < 0.05, ** *p* < 0.01 in comparison with their own D7 data in (**C**). *** *p* < 0.001 in comparison to D7 data in (**D**). One-way ANOVA followed by Dunnett test.

Across different species and tissue conditions, our results have shown that Flt3 expression in cerebellum is restricted to the cell body and processes of Purkinje cells and other neurons. This neuron-specific expression pattern suggests a potential specialized role for Flt3 in cerebellar function and development. Building on the finding of neuron-specific expression of Flt3, we employed the AP-IHC hybrid method to investigate the temporal dynamics of Flt3 expression during mouse brain development. Our analysis revealed a significant increase in Flt3 immunoreactivity within the cerebellum from postnatal day seven to two months of age, suggesting a developmental upregulation of *Flt3* gene expression (Figure 4A,B). Importantly, the subcellular localization of Flt3 underwent a developmental shift. At early postnatal stages, Flt3 was predominantly cytosolic, while in the mature brain, a substantial proportion of the protein was observed in dendrites, indicating potential developmental changes in its functional roles (Figure 4). Thus, the AP-IHC hybrid method has allowed us to establish, for the first time, the brain region- and cell-type-specific expression pattern of Flt3, which aligns closely with previously reported single-cell sequencing data. Furthermore, this approach enabled the in situ detection and quantification of Flt3 subcellular localization across developmental stages, uncovering a dynamic cytosol-to-dendrite redistribution in cerebellar Purkinje cells, laying the foundation for future research on the role of Flt3 in cerebellar function and its implications for brain health and disease.

### 2.2. AP-IHC Immunolabeling of Potassium Channel Kir2.1 in Mouse, Pig, and Human Brain Tissues

**Figure 5:**
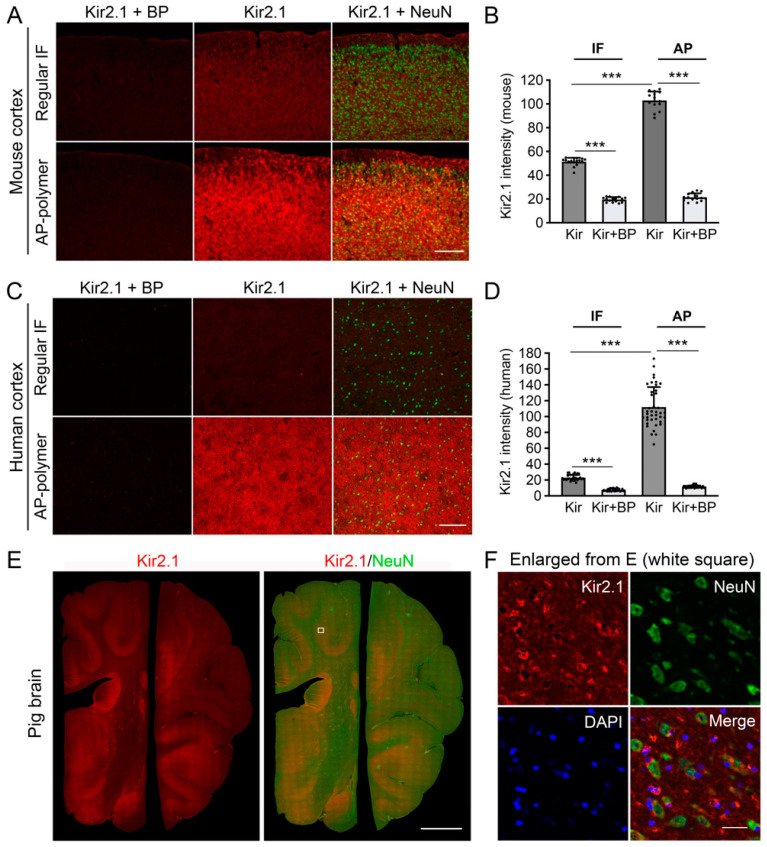
The AP-IHC method robustly labels Kir2.1 protein expression in mouse, pig, and human brain tissues. Kir2.1 is one of the potassium channels on the cell membrane and sometimes not easy to see clearly by the regular IF staining. The regular IF staining and the AP-IHC hybrid staining were compared respectively in the mouse cortex (**A**,**B**) and human cortex (**C**,**D**). All staining was to co-label Kir2.1 with NeuN, and the staining control was processed exactly the same as the others, except the Kir2.1 primary antibody was pre-incubated with a Kir2.1 blocking peptide (Kir2.1+BP) 30 min before being applied onto the sections. Quantification of the staining intensity vs. its own control (Kir vs. Kir+BP) is summarized in (**B**,**D**), Mean ± SD. *** *p* < 0.001. One-way ANOVA followed by Bonferroni correction. The AP-IHC hybrid method was also used on pig brain sections (**E**, stitched image), and the enlarged cellular images are shown in (**F**). Scale bars in (**A**): 450 µm; (**B**): 400 µm; (**E**): 2.5 mm; (**F**): 30 µm.

To further evaluate the versatility of the AP-IHC immunolabeling method, we explored its application in detecting Kir2.1, a voltage-gated inward-rectifying potassium channel, in brain tissue. Kir2.1 plays a crucial role in regulating the neuronal resting membrane potential but is expressed at low levels in the brain, complicating its detection [13]. Moreover, its cell-type-specific expression patterns in the brain remain poorly characterized. We compared Kir2.1 immunostaining in mouse brain tissue using either the regular fluorescent immunolabeling or the AP-IHC method. The AP-IHC method demonstrated substantially improved labeling sensitivity and signal-to-noise (S/N) ratio (Figure 5A,B). To ensure high specificity, negative control experiments were conducted by pre-incubating the Kir2.1 primary antibody with an excessive amount of Kir2.1 blocking peptide (Kir2.1+BP) to neutralize the antibody for 30 min before applying it to tissue sections (see Method Section for details) [14]. Expanding beyond mouse tissue, we applied the same AP-IHC immunolabeling protocol to human (Figure 5C,D) and pig cortical sections (Figure 5E,F). Consistent staining patterns across species were observed, underscoring the method’s robustness. High resolution microscopy revealed that Kir2.1 staining was localized around neurons, co-labeled with the neuronal marker NeuN (Figure 5F), suggesting that Kir2.1 expression is predominantly neuronal in the cortex. This study highlights the AP-IHC method as a highly sensitive and specific tool for detecting low-abundance membrane proteins such as Kir2.1 across species, providing new insights into their cellular and subcellular distribution in the brain.

### 2.3. AP Immunolabeling of PSD95 Within the Synapses in Human Stem Cell-Derived Neurons

**Figure 6:**
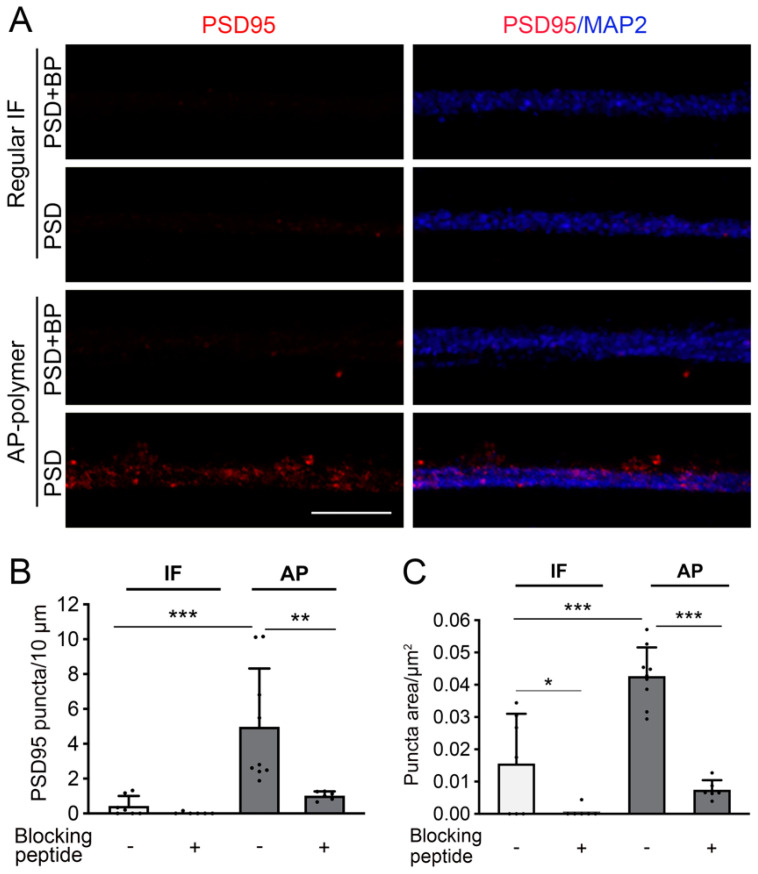
AP-IHC hybrid staining method is suitable for fine structures in dendritic spines. Human iPSC-neurons were cultured for 5 months and stained with PSD95 (red) and MAP2 (blue) antibodies by the AP-IHC hybrid staining method. The regular immunofluorescent staining was used for a comparison (Regular IF). The staining control was carried out using the PSD95 antibody pre-incubated with its blocking peptide (PSD95+BP) to replace the PSD95 antibody. Images were taken with the Zeiss LSM 980 airyscan 2 confocal microscope (63× object). While almost no signals were seen from the regular IF staining, the hybrid method provides bright staining in synaptic puncta pattern along the dendrite (**A**). The visible puncta numbers per 10 µm were quantified in (**B**) and the average puncta area per µm^2^ were shown in (**C**). Scale bar in (**A**): 5 µm. Mean ± SD, * *p* < 0.05; ** *p* < 0.01; *** *p* < 0.001 in (**B**,**C**). One-way ANOVA followed by Tukey test.

Building on the success of the AP-IHC hybrid immunolabeling method for visualizing cellular resolution protein localization, we extended its application to examine fine subcellular structures, particularly synapses. Using cultured human-induced pluripotent stem cell (iPSC)-derived neurons, which are widely applied in disease modeling and in drug screening studies [7,15], we explored the labeling of the synaptic scaffold protein PSD95 using the AP-IHC method. Our prior work demonstrated the functional maturation of synapses in human iPSC-derived neurons co-cultured with astrocytes [16], yet regular immunofluorescent staining cannot adequately visualize PSD95 in human neurons (unpublished results and private correspondence). In this study, MAP2 antibody was used to mark neuronal dendrites, while PSD95 staining was performed using either the standard immunofluorescence method [17] or AP-IHC hybrid immunolabeling. Results showed that, compared to the standard immunostaining, the AP-IHC method results in robust labeling of discrete PSD95-positive synaptic puncta along the MAP2^+^ dendrites (Figure 6). The specificity of staining was confirmed by pre-incubating the PSD95 antibody with its blocking peptide (PSD95+BP), which diminished the signal (Figure 6). These findings highlight the potential uses of AP-IHC immunolabeling to investigate fine structures such as synapses with high sensitivity and specificity. This methodology represents a powerful tool for neuroscience research, enabling detailed studies of the cellular and subcellular distribution of important neuronal antigens.

## 3. Discussion

This study offers the first comprehensive characterization of the receptor tyrosine kinase Flt3’s expression pattern during brain development. The Flt3 molecule is well-studied in the context of hematology, particularly for its role in leukemia, where constitutive phosphorylation due to mutations drives oncogenesis [18]. A number of Flt3 inhibitors have been developed for leukemia treatment [19]. However, its roles in the brain remain largely unexplored, primarily due to the challenges in detecting its protein expression at the cellular resolution. Recent findings have linked Flt3 signaling to neurological processes. Pharmacological inhibition of Flt3 has been associated with increased KCC2 expression, a chloride transporter critical for GABAergic inhibition [7], which indicates potential treatment of neurological disorders. A recent study shows that Flt3 is expressed in the Purkinje cells in the cerebellum tissue [5]. Using our optimized hybrid AP-IHC immunolabeling method, we found that the Flt3 protein in the cerebellum is predominantly expressed in the GABAergic Purkinje neurons. During cerebellar development, Flt3 localization in Purkinje cells shifts from primarily cytosolic at early stages of brain development to a dendrite-enriched pattern in the molecular layer of mature brains. These findings align with previous evidence that Flt3 modulates neuronal gene expression and highlight its potential involvement in brain development and neurological diseases. This study not only provides strong evidence to resolve the difficulties of visualizing Flt3’s neuronal expression patterns but also lays a foundation for further research into its functional roles in the brain. These insights may open new avenues for leveraging Flt3-targeting therapeutics in neurodevelopmental and neurodegenerative disorders.

Through this study, we developed a hybrid method combining the high sensitivity of AP-polymer-based histochemistry with standard immunofluorescent staining, enabling robust co-staining of multiple markers. HRP histochemistry, while highly sensitive and useful in detecting proteins expressed at low levels, has traditionally been limited by the difficulty of co-staining, which is often required for mechanistic investigation. This limitation arises because standard histochemical reactions yield chromogenic products, such as brown (DAB substrate) or blue (hematoxylin), which overlap and cannot be separated effectively for multiplex analyses [20]. Additionally, the color palette is inherently restricted. The AP-IHC methodology overcomes these limitations by offering two key advantages: (1) enhanced sensitivity: the use of micro-polymer technology significantly amplifies staining sensitivity, making it highly effective for low-abundance proteins. (2) High fluorescent output: the fluorescent substrate of AP enables compatibility with fluorescence microscopy, facilitating multiplexed visualization. Our hybrid method utilizes a sequential staining protocol, where AP polymer histochemistry labels weakly expressed proteins, while immunofluorescent staining is applied to mark additional antigens. The fine tuning of detergent usage, as detailed in the Methods section, is crucial for the success of this technique. This approach allows researchers to visualize distinct fluorescent signals with different wavelength filters, bypassing the limitations of traditional chromogenic co-staining methods. We anticipate that this hybrid method will be a valuable tool for the research community, offering a practical solution for studies requiring high sensitivity and multiplexed analysis of protein expression.

A potential limitation of the AP-IHC method is that it may not effectively label all low-abundance proteins. Therefore, ensuring staining specificity should always be a top priority. To minimize the risk of false-positive signals, a negative control should always be included in parallel with each staining experiment. Ideally, the negative control should be on the same glass slide, adjacent to the sections stained with specific antibodies, to ensure identical processing conditions. An isotype-matched IgG negative control should be used as the negative control instead of simply omitting the primary antibody. Only the staining signals significantly above the negative control can be considered positive and specific. Besides specificity, it is also important to assess whether AP-IHC staining capabilities extend beyond the rabbit host reagents used in this study. Additionally, exploring AP-IHC’s compatibility with hybrid co-staining, such as proposed in this study, could expand its utility for research.

In this study, we demonstrated the feasibility and versatility of applying a highly sensitive AP-IHC plus conventional immunofluorescence staining method to label a variety of proteins crucial to neuronal function. Using this method, we achieved detailed immunolabeling of the developmental trajectories and protein localization at cellular and subcellular resolutions for Flt3 and several other key neuronal proteins. We established the neuron-enriched expression pattern of the inward-rectifying potassium channel Kir2.1 in mouse brain tissue, a feature conserved in pig and human brain tissues. This cross-species consistency underscores its functional relevance and provides a cellular basis for targeting Kir2.1 in therapeutic interventions. The AP-IHC hybrid immunolabeling method proved capable of resolving synaptic-scale structures by labeling the post-synaptic density protein PSD95 in human stem cell-derived neurons. This advancement opens new possibilities for studying synapse-level organization and protein interactions, an area critical to understanding neuronal connectivity and plasticity [21]. The AP-IHC hybrid immunolabeling method demonstrates broad applicability across various sample types, including mouse, pig, and human brain tissue as well as cultured human stem cell-derived neurons. Its ability to enhance detection sensitivity while resolving complex subcellular structures makes it a powerful tool for neuroscience research. Our results underscore the utility of in situ protein detection techniques, such as AP immunolabeling, for bridging the gap between transcriptomic data and protein-level insights. By validating RNA sequencing results and providing spatial and functional context for the protein products of genes of interest, this approach offers a powerful complement to existing genomic tools and datasets. It enables precise mapping of protein localization and dynamics within neuronal systems, thereby advancing our understanding of brain development and synaptic organization. Furthermore, this method holds significant potential for identifying and characterizing therapeutic targets in translational neuroscience research.

## 4. Materials and Methods

### 4.1. Animals

Normal wild-type C57BL/6J mice (0–2 months old, Jackson labs, Bar Harbor, ME, USA: Strain #:000664) were used for generating the mouse brain tissue slices. Animal housing and perfusion procedures for tissue harvesting followed the policies at Boston Children’s Hospital (BCH) and were approved by the BCH Institutional Animal Care and Use Committee.

### 4.2. Tissue Sample Preparation

Mouse, pig, and human brain tissue sections were used in this study. Mouse brain sections were primarily used to compare three different immunostaining methods (described below), establish the AP-IHC method and hybrid co-immunostaining methods, and examine the expression pattern of the Flt3 protein during brain development. Pig and human brain sections were included to evaluate the applicability of the staining method across different species and tissue sample storage/processing conditions.

For the mouse brain section preparation, mice were euthanized by overdose of isoflurane and neck dislocation, and perfused transcardially with cold saline (0.9% NaCl) followed by 4% paraformaldehyde (PFA) at all required ages. The perfused brains were post-fixed in 4% PFA for 2 days at 4 °C, then changed to 30% sucrose at least twice until cryostat sectioning. For most studies, the O.C.T.-embedded mouse brains were cut at 10 µm in thickness sagittally using the Leica cryostat machine (Leica CM3050S, Leica Biosystems, Deer Park, IL, USA) and the sections were directly mounted onto the VWR superfrost glass slides (see Supplementary Table S3). In the floating staining experiment, the brain blocks were cut at 40 µm in thickness and the slices were collected in PBS tubes. Paraffin-embedded pig brain coronal sections were acquired from Dr. Jianhua Qiu in the Mannix lab, Boston Children’s Hospital. Formalin-fixed human brain tissues were acquired from the BCH pathology department and processed in the Pathology Core of Beth Israel Hospital for paraffin embedding and sectioning (5 µm in thickness). Two brain sections were mounted on each glass slide.

The sample size and usage principle for each experiment depended on the specific goals of the study. Following the ARRIVE guidelines 2.0, we provide detailed information for each experiment below.

Figure 1: The objective was to compare 3 different staining methods. In each round of staining, we used neighboring mouse brain sections to ensure identical tissue conditions, with the only variable being different staining methods. Images were taken from the same cerebellar region to facilitate direct comparison. Data from Figure 1 were obtained from three independent rounds of staining using brain sections from three adult mice.

Figure 2: This experiment aimed to establish the hybrid co-staining method and examine the cellular expression pattern of Flt3. Sections from four postnatal D14 mouse brains (equal distribution of males and females) were used.

Figure 3: Human brain sections were obtained from a single tissue block, and multiple rounds of staining were conducted to validate AP-IHC performance and test the co-staining with other antibodies, as many antibodies were not working well in long-term fixed human brain samples (Figure 3A). In Figure 3B, thick brain sections were sourced from the same animals in Figure 2. The goal was to demonstrate that the AP-IHC is effective in floating section staining.

Figure 4: This experiment quantitatively assessed Flt3 expression during development. We analyzed Flt3 expression at postnatal D7, D14, D21, and D60, using 3–4 mice per time point (approximately equal male-to-female ratio), totaling 15 mouse brains. In each brain, we chose at least 3 pairs of cerebellum sections >100 µm apart, resulting in 15 × 3 = 45 pairs of sections for the staining. Within each pair, one section was stained for Flt3+NeuN, while the adjacent section on the same glass slide served as a negative control (for measuring the staining baseline). In each stained cerebellum section, including both the negative control and specific Flt3 staining, 3–4 different areas were imaged, and their average intensity was considered as a representative. In all of the sections’ imaging processes, the exposure time was kept same and was initially determined by the most suitable time for the sections stained Flt3 with the highest fluorescence to avoid overexposure, then this time was applied in all imaging. The Flt3 intensity on each glass slide was first averaged by its own 3 areas, then normalized by the negative control of the neighboring section, and finally counted into the groups for the statistical analysis.

Figure 5: This experiment tested the AP-IHC method’s ability to detect Kir2.1, a challenging protein to stain for. In Figure 5A,B, motor cortex sections from three adult mice (same animals as the D60 group in Figure 4) were used for staining comparison. As in Figure 1, neighboring section pairs were selected to compare standard immunofluorescent staining with AP-IHC. Each pair included one section stained for Kir2.1+NeuN and another control section that underwent the same staining in the presence of the Kir2.1 antibody blocking peptide. Figure 5C,D compared human cortex sections all sourced from a single tissue block. Figure 5E,F were from a single pig brain.

Figure 6: All samples were from cultured human iPSC-derived neurons. Coverslips from the same culture batch were stained simultaneously to compare different staining methods for detecting PSD95, a protein well known to be difficult to detect through immunostaining in human neurons.

### 4.3. Human Stem Cell-Derived Neuron Culture and Cell Sample Preparation

Human embryonic stem cell (hESC)-derived neural progenitor cells (NPCs, generated from the WIBR1 hESC line) were seeded at 10,000 cells per well onto glass coverslips pre-seeded with an astrocyte feeder layer [16] and cultured for 5 months for PSD95 staining.

### 4.4. Immunolabeling

Three different immunolabeling methods were used in this work: the regular immunofluorescent staining, HRP-based histochemistry, and the AP polymer-based histochemistry hybrid with the regular fluorescent staining. All information on primary antibodies, secondary antibodies, as well as other key materials and reagents are summarized in Supplementary Tables S1–S3, respectively.

1. Regular immunofluorescent staining The cryostat brain sections were heated on a heating plate for 20 min (37–42 °C) to dry out the wetness from the freezer, and O.C.T. was washed off in Tris-buffered saline (TBS). The pig and human brain paraffin-embedded sections went through xylene and a series of different concentrations of ethanol and were washed in TBS. All brain slides went through antigen retrieval at 95–100 °C for 10 min, then incubation with the blocking buffer (5% serum + 2% BSA in TBS-T (0.1% Tween-20)) for 1 h at room temperature, primary antibody incubation overnight at 4 °C and fluorescent secondary antibodies, and were then coverslipped with DAPI-fluoromount mounting medium.
2. HRP-IHC Similar to the regular immunofluorescent staining, the brain section slides went through antigen retrieval and were incubated with 0.1% H_2_O_2_ to inactivate the endogenous HRP. After blocking, one section on each slide was incubated with the Flt3 primary antibody at 4 °C overnight and the other section on the same slide was used as the staining control, which used the same amount of rabbit IgG to replace the Flt3 rabbit antibody. On the second day, after the TBS-T wash, the slides were incubated with goat anti-rabbit biotinylated secondary antibody, followed by VECTASTAIN ABC Reagent, and developed in freshly made DAB peroxidase substrate solution. Monitor the color development under the microscope until satisfied by comparing with the control section on the same slide. The stained slides went through a series of ETOH and Xylene, and were coverslipped with Cytoseal.
3. AP-IHC hybrid with regular fluorescent immunostaining For the co-staining of Flt3 (rabbit) with NeuN (mouse), GFAP (chicken), Iba1 (goat), parvalbumin (chicken), and calbindin (goat) in brain sections, we used AP-IHC to boost Flt3’s signal and co-stained with other cell specific markers. The brain slides were processed the same as in the above HRP-IHC method (except we omitted the H_2_O_2_ step) until adding the secondary antibody. Instead of the biotinylated secondary antibody in HRP histochemistry, the horse anti-rabbit AP-conjugated secondary antibody was used for 10 min at room temperature. After a brief wash with TBS-T and TBS, we developed AP with the substrate solution, monitored the color under the microscope, and stopped the reaction by an immediate TBS wash. AP-IHC produced the red fluorescent precipitance (wavelength similar to Alexa 594). The brain slides continued to be incubated with other fluorescent secondary antibodies for the co-stained fluorescence (e.g., Alexa 488, 647, etc.), and after brief washes, were then coverslipped with DAPI-fluoromount mounting medium.

For the thick brain slices, the floating staining was carried out in the Eppendorf tubes or in the 24-well-plate wells. The same procedures as for the slice-mounted slides were used, except the high-pH washing buffer TBS-T or TBS (made from Trizma base without adjusting pH), in order to minimize the background. After the co-staining was finished, we mounted the brain slices onto the glass slides and coverslipped them with DAPI-fluoromount mounting medium. The negative control tube was also set up and parallel-processed in each staining.

For cultured cell staining, the procedures were the same as above, except using PBS-T (0.2% Triton X-100) and PBS to replace TBS-T and TBS. The negative control cell coverslip was also set up and parallel-processed in each staining.

There are several critical points to make a successful co-staining with this hybrid method: (1) after the AP-IHC color development, no detergent buffer is allowed to be used in the subsequent co-staining procedures, since the detergent destroys the precipitate pattern from AP-IHC. (2) Always have a negative staining control with the same amount of IgG to replace the primary antibody on the other brain section of the same glass slide. (3) Similar to the HRP histochemistry, endogenous AP inactivation should be considered. Heating slides (like antigen retrieval) is a good way to inactivate the endogenous AP. If the staining protocol does not include heating, an AP inhibitor (Levamisole) may be considered.

### 4.5. Imaging and Quantification

With our strict principle for the control setting in each staining of every single slide (two sections on one slide; using same amount of IgG to replace the antibody; using blocking peptide to pre-incubate with antibody, exact same development time, etc.), images from the positive staining and the control staining were always taken with the exact same setting for exposure conditions. Most images in this study were taken under a regular fluorescent microscope (10×) and some of them (floating staining of thick brain slices and PSD95 synapse staining images) were taken with the Zeiss LSM 980 w/Airyscan 2 confocal microscope (10× for thick slice imaging, 63× for PSD95 synapse imaging, Oberkochen, Germany). For each of the large pig brain slices, a few hundred images were taken and stitched together automatedly with the Zeiss Axio Imager Z2 microscope (Oberkochen, Germany).

For the staining intensity quantification, the single channel images and other co-stained channel images were first merged together with Image J 3 (1.53K) in order to draw the outlines of the exact areas for the quantification. After outlined the measuring areas, the merged images were split the colors and only the target channel (for example, the red channel of Flt3) was used to measure the intensity. On each glass slide, the measured intensity data were first normalized by their own staining control data from the other section on the same slide, then they were included in the groups. The detailed sample numbers depended on the different experiments and are described above in the sample size part. PSD95 puncta quantification was carried out with FIJI (version 1.0) puncta measurement procedure.

All images generated from microscopes and graphs from GraphPad Prism 10 (version 10.2.3) or Microsoft Excel (version 16.94) were assembled with Adobe Photoshop 2024.

### 4.6. Statistics

GraphPad Prism 10 was used for all statistical analysis. Detailed analysis is described in each figure legend.

## Data Availability

The original contributions presented in this study are included in the article/Supplementary Material. Further inquiries can be directed to the corresponding author.

## Supplementary Materials

The supporting information can be downloaded at https://www.mdpi.com/article/10.3390/ijms26052284/s1.
