# Supplementary material for: Visualizing the Cellular and Subcellular Distribution of Fms-like Tyrosine Kinase 3 (Flt3) and Other Neuronal Proteins Using Alkaline Phosphatase (AP) Immunolabeling
Source: Int J Mol Sci. 2025 Mar 4;26(5):2284. doi: 10.3390/ijms26052284 (PMC11900488; doi:10.3390/ijms26052284)
Supplement: Supplementary file 1 [file ijms-26-02284-s001.zip › ijms-3469240-supplementary.pdf]

Supplementary Figure S1.

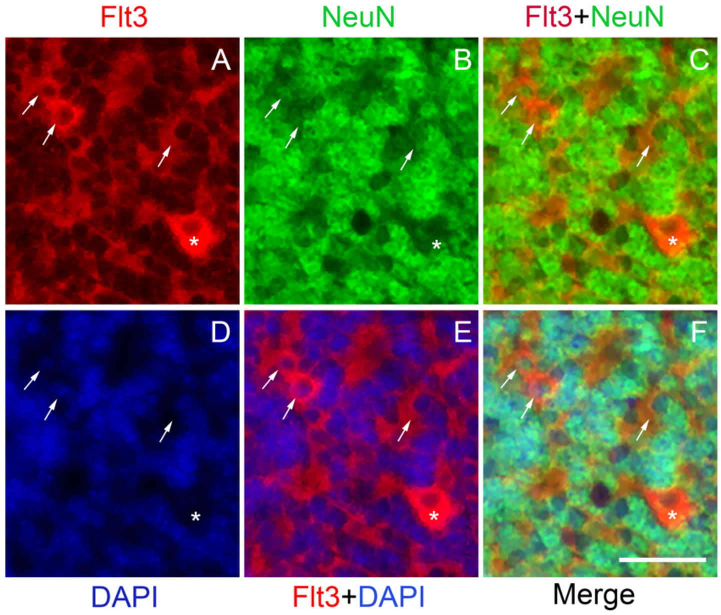

Supplementary Figure S1: Flt3 expression in the granular layer is enriched in NeuN$^+$ cells. While the majority of Flt3 (red) is expressed in Purkinje cells (PCL) and their processes in the molecular layer (ML) described in Figure 2, some Flt3$^+$ cells are present in the granular layer (GL, arrows in **A-F**). The majority of these cells were co-labeled with NeuN immunostaining (green, **B**). Several large, sparsely distributed cells (*) exhibited high levels of Flt3 expression but did not co-localize with NeuN, similar to the pattern observed in Purkinje cells. Scale bar: 50 µm.

Supplementary Figure S2.

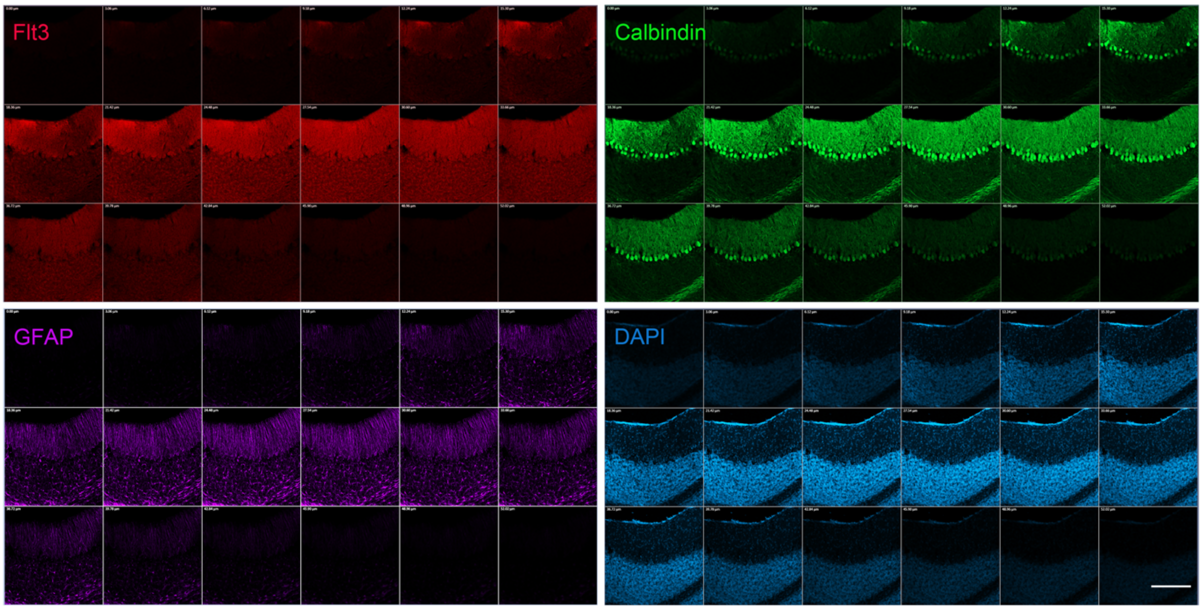

Supplementary Figure S2: Flt3 AP-polymer IHC staining was distributed throughout the entire thickness of the brain slice. Mouse cerebellum was cryostat sectioned at 40 μm in thickness, and the hybrid floating staining method was used to visualize Flt3 (red) alongside other cellular markers calbindin (green), GFAP (purple), and DAPI nucleic labeling (blue). Imaging was performed using a Zeiss LSM980 Airyscan confocal microscope with scanning a total thickness of 52.02 μm at 3.06 μm intervals, yielding a series of 18 scanned levels. Flt3 staining was consistently distributed across all tissue-containing levels, with the maximal signal intensity observed in the central layers (the 7th-13th levels), indicating that the staining from AP-IHC is not limited to the tissue surface even in thick sections. In addition, AP-IHC Flt3 staining did not interfere with other co-stained markers, as the Calbindin, GFAP and DAPI staining exhibited the same strongest signal levels as that in AP-IHC Flt3. Scale bar: 200 μm.

Supplementary Table S1. Summary of primary antibodies

**Primary antibody (target)**	**Vendor**	**Catalog number**	**Host species**	**Dilution ratio**
Calbindin	Invitrogen	PA5-46936	Goat	1:200
Flt3	Abclonal	A12462	Rabbit	1:200
GFAP	Aves Labs	GFAP	Chicken	1:2000
Iba1	Abcam	Ab5076	Goat	1:100
Kir2.1	Alomone Labs	APC-026	Rabbit	1:200
Kir2.1 blocking peptide	Alomone Labs	BLP-PC026	Synthetic	>= Kir2.1
MAP2	EnCor	MCA-4H5	Mouse	1:500
MAP2	Millipore Sigma	AB15452	Chicken	1:1000
NeuN	Invitrogen	MA5-33103	Mouse (1B7)	1:2000
NeuN (for human tissue)	Invitrogen	PA5-143586	Goat	1:1000
Parvalbumin	Invitrogen	PA5-143579	Chicken	1:1000
PSD95	Alomone Labs	APZ-009	Rabbit	1:400
PSD95 blocking peptide	Alomone Labs	BLP-PZ009	Synthetic	>= PSD95
TUJ1 (bIII tubulin)	BioLegend	801202	Mouse	1:500

Supplementary Table S2. Secondary antibodies used in this study

Secondary antibody	Vendor	Catalog number	Dilution ratio
Alexa Fluor 488/594/647 different species secondary antibodies	Invitrogen	Depend on color and species	1:500
Goat-anti-rabbit biotinylated	Vector Laboratories	BA-100	1:500
ImmPRESS-AP Horse-anti-rabbit IgG kit	Vector Laboratories	MP-5401	Ready to use

Supplementary Table S3. Other key materials & reagents

Reagent/material name	vendor	Catalog number	Usage note
Antigen unmasking solution	Vector Laboratories	H-3300-250	1:100 diluted in H_2O
DAPI-fluoromount-G	Southern Biotech	0100-20	Follow instructions
DAB substrate kit	Vector Laboratories	SK-4100	Follow instructions
ChromPure Rabbit IgG, whole molecule	Jackson Immunoresearch Labs	011-000-003	Use as staining control for rabbit antibody in AP-IHC
Fluoromount-G	Southern Biotech	0100-01	Follow instructions
ImmPACT Vector Red Substrate kit (AP)	Vector Laboratories	SK-5105	Follow instructions
Premium Superfrost Plus microscope slides	VWR	48311-703	N/A
VECTASTAIN ABC-HRP kit	Vector Laboratories	PK-6100	Follow instructions
